# Dr. Edward Mulheron

**Published:** 1900-05

**Authors:** 


### OBITUARY.

DR. EDWARD MULHERON, of Binghamton, N.Y., died at his residence in that city April 6, 1900, aged 53 years. He graduated at the Medical Department of the University of Buffalo in 1872.

